# *Babesia* BdFE1 esterase is required for the anti-parasitic activity of the ACE inhibitor fosinopril

**DOI:** 10.1016/j.jbc.2023.105313

**Published:** 2023-10-04

**Authors:** Pratap Vydyam, Jae-Yeon Choi, Shalev Gihaz, Meenal Chand, Meital Gewirtz, Jose Thekkiniath, Stefano Lonardi, Joseph C. Gennaro, Choukri Ben Mamoun

**Affiliations:** 1 Section of Infectious Diseases, Department of Internal Medicine, Yale School of Medicine, New Haven, Connecticut, USA; 2 Department of Computer Science and Engineering, University of California, Riverside, California, USA

**Keywords:** human babesiosis, babesia, parasite, *Babesia duncani*, FDA-approved drugs, fosinopril, fosinoprilat, BdFE1

## Abstract

Effective and safe therapies for the treatment of diseases caused by intraerythrocytic parasites are impeded by the rapid emergence of drug resistance and the lack of novel drug targets. One such disease is human babesiosis, which is a rapidly emerging tick-borne illness caused by *Babesia* parasites. In this study, we identified fosinopril, a phosphonate-containing, FDA-approved angiotensin converting enzyme (ACE) inhibitor commonly used as a prodrug for hypertension and heart failure, as a potent inhibitor of *Babesia duncani* parasite development within human erythrocytes. Cell biological and mass spectrometry analyses revealed that the conversion of fosinopril to its active diacid molecule, fosinoprilat, is essential for its antiparasitic activity. We show that this conversion is mediated by a parasite-encoded esterase, BdFE1, which is highly conserved among apicomplexan parasites. Parasites carrying the L238H mutation in the active site of BdFE1 failed to convert the prodrug to its active moiety and became resistant to the drug. Our data set the stage for the development of this class of drugs for the therapy of vector-borne parasitic diseases.

Human babesiosis is a rapidly emerging tick-borne infectious disease caused by apicomplexan intra-erythrocytic parasites of the genus *Babesia* (1). The rising trend in the number of human babesiosis cases among both immunocompromised and immunocompetent individuals is a call for concern (1, 2). Among the eight *Babesia* species known to cause human babesiosis worldwide, *Babesia microti* is responsible for most clinical cases reported to date and is considered endemic in the United States (1, 3). Other cases of human babesiosis include *Babesia divergens* in Europe and *Babesia duncani* in the Western United States (1, 4). The increase in the geographic distribution of the tick vectors, which has been influenced by the environmental changes of the last decades and various anthropogenic factors, is considered the main driver of the recent increase in tick-borne infections. Over 16,000 cases of human babesiosis in the United States were reported to the CDC between 2011 and 2019 (5). Annual cases more than doubled during this period, with some northeastern states experiencing case growth above 100% (5). Current therapies recommended for the treatment of human babesiosis consist of two combinations of antimalarial drugs: quinine + clindamycin recommended for the treatment of severe disease and atovaquone + azithromycin for the treatment of mild babesiosis cases. However, both combinations are associated with mild or severe adverse events. Furthermore, several cases of treatment failure caused by the emergence of parasites resistant to atovaquone or azithromycin have been reported (6, 7). This has spurred investigation into repurposing other FDA-approved drugs such as proguanil and tafenoquine for the treatment of babesiosis as well as pre-clinical evaluation and development of novel anti-babesiosis drugs (1).

Past efforts to understand the biology of *Babesia* parasites that infect humans and to develop new therapies that are specifically tailored to inhibit their growth were limited by the lack of a suitable *in vitro* culture model. This challenge was recently surmounted following the development of a continuous *in vitro* culture system of *B. duncani* in human red blood cells (hRBCs) paired with an optimized mouse model of lethal infection. This model, dubbed the In Culture–In Mouse model of *Babesia* infection, has been instrumental in advancing *Babesia* biology, genomics, cell biology, diagnostics, and drug discovery (8, 9, 10).

Among the most widely used drugs for the treatment of human illnesses, angiotensin-converting enzyme (ACE) inhibitors, which inhibit the ACE-1 enzyme, have unique physicochemical properties and excellent safety profiles (11). The compounds are estimated to be used by more than 20% of adults globally for the treatment of hypertension and heart failure (12). Unlike ACE-2 receptor, the human receptor for the SARS-CoV2 virus (13), ACE-1 is a soluble di-peptidyl carboxypeptidase enzyme and catalyzes the conversion of the 10-amino acid angiotensin-I (Ag-I) to the 8-amino acid angiotensin-II (Ag-II) (14, 15). Several classes of ACE inhibitors have been developed over the years, the first of which was the sulfhydryl-containing compound captopril, which was released commercially in 1981 as an orally active ACE inhibitor. Another ACE inhibitor with more favorable pharmacological properties due to its low incidence of adverse events is fosinoprilat, an active phosphonic acid metabolite of the prodrug fosinopril. The latter is rapidly hydrolyzed in the liver and intestine into fosinoprilat with peak plasma concentration attained within 3 h after prodrug ingestion (16, 17). While these drugs have been massively used worldwide as ACE inhibitors, no prior use of these molecules as antiparasitic has been demonstrated heretofore.

Here we report the first evidence for the activity of fosinopril as a potent antibabesial drug with efficacy *in vitro* in the nanomolar range. We show that the prodrug is rapidly taken up by *B. duncani*-infected erythrocytes and subsequently converted by a parasite-specific esterase into its active molecule, fosinoprilat.

## Results

### Identification of fosinopril as a potent antibabesial drug

**Figure 1:**
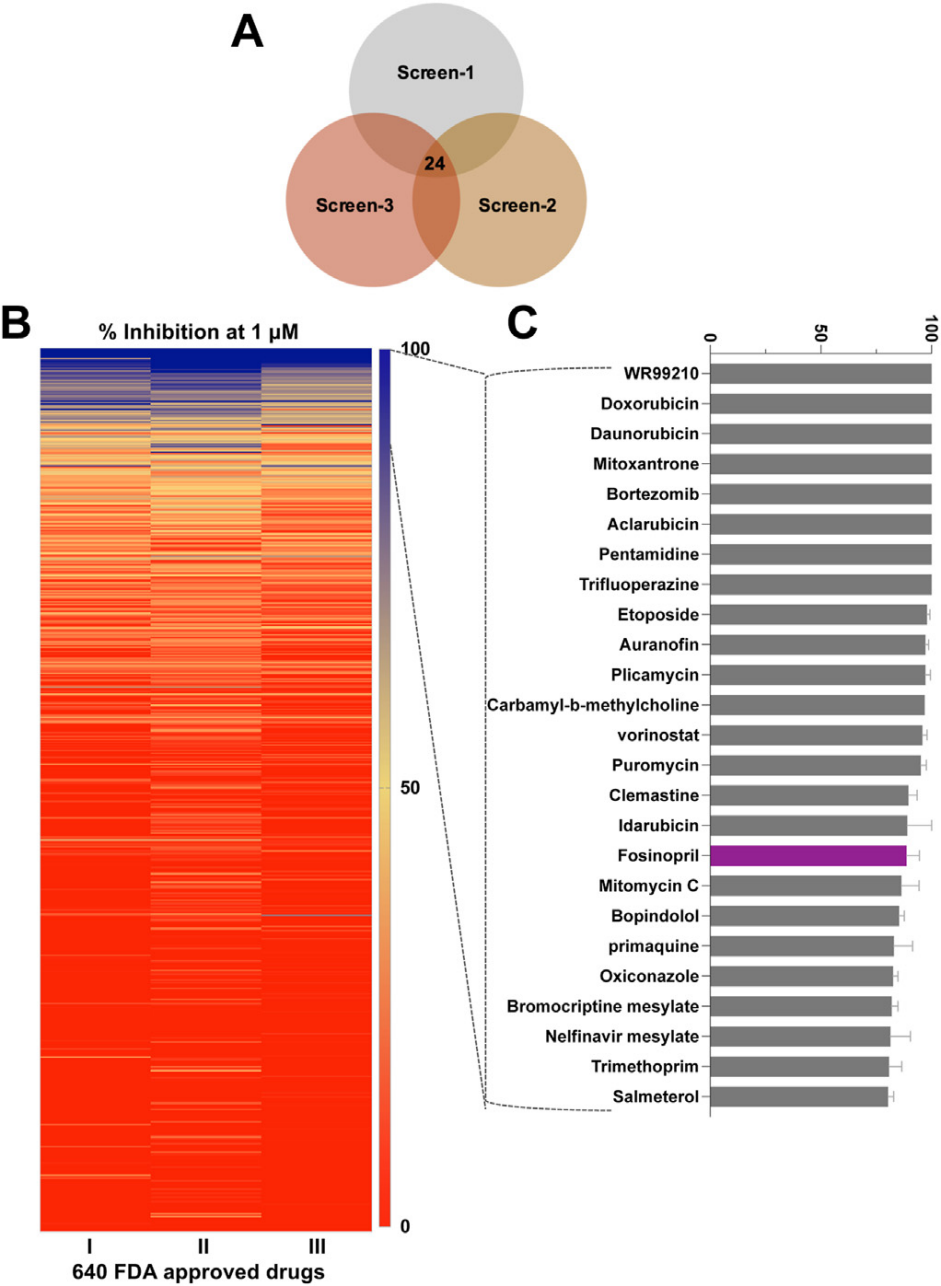
**Screen of 640 FDA-approved drugs against *B. duncani* parasite growth *in vitro* and shortlist compounds with effective antibabesial activity.***A*, Venn diagram of the primary screen design, comprising three independent screens. Drugs that inhibited growth by 80% or more were considered hits, and drugs that were hits in all three screens were shortlisted for further study. *B*, heat map illustration of the percent inhibition results for three independent screens (I, II, and III) that were conducted on 640 FDA-approved drugs at 1 μM. Values represent percent *B. duncani* growth inhibition, ranging from *red* (no inhibition) to *blue* (100% inhibition). *C*, shortlisted drugs exhibiting >80% *B. duncani* growth inhibition in each of the three screens. The ACE inhibitor fosinopril is highlighted in *purple*. Each data point on the graph represents the mean of three independent experiments ±SD.

**Figure 2:**
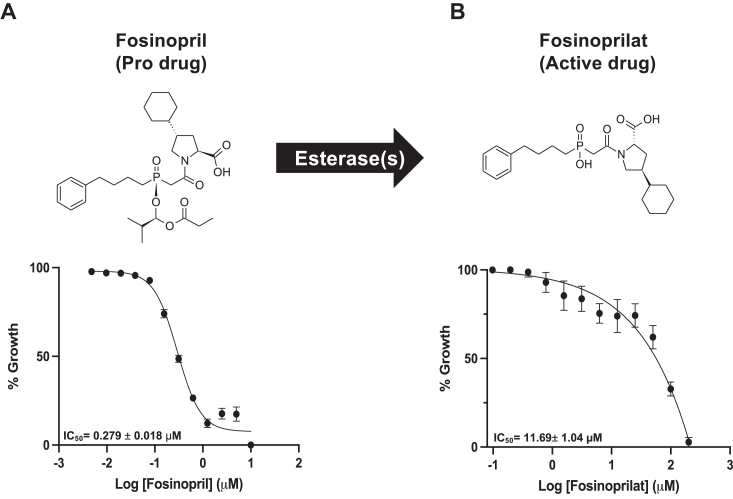
**Esterification of fosinopril to its active drug fosinoprilat and efficacy against *B. duncani* parasite.***A*, chemical structure of fosinopril, an ACE-1 inhibitor pro-drug, and dose-response curve of *B. duncani* growth as a function of fosinopril concentration revealing IC_50_ of 0.279 ± 0.018 μM. *B*, chemical structure of fosinoprilat, the active drug formed through the esterification of fosinopril by either host or parasite esterase, and dose–response curve of *B. duncani* growth as a function of fosinoprilat concentration revealing IC_50_ of 11.69 ± 1.04 μM. The mean IC_50_ values were determined and represented on the graph along with the corresponding standard deviation (IC_50_ ± SD). Each data point is the average of three independent experiments, each with biological triplicates.

The development of the *B. duncani* continuous *in vitro* culture in hRBCs (Supplementary Information) and assays for rapid measurement of parasite proliferation *in vitro* (8, 18, 19) made it possible to screen a library of FDA-approved drugs for antibabesial activity. Three independent screens of the library were conducted at a concentration of 1 μM of the target compounds (Fig. 1*A*). Untreated parasites or those treated with the antifolate WR99210 at 1 μM, which results in 100% inhibition, were used as controls (Fig. 1*B*). Of the 640 FDA-approved drugs screened, 24 compounds inhibited parasite proliferation by more than 80% at 1 μM in each of the three replicates (Fig. 1*C*). Among these, the ACE-1 inhibitor fosinopril was selected as an ideal drug because of its high *in vitro* efficacy (>95% inhibition at 1 μM), its well-known pharmacological properties, and its well-established safety profile. To further evaluate the potency of the compound, dose–response assays were conducted using fosinopril as well as its known active drug fosinoprilat. While the half-maximal inhibitory concentration (IC_50_) of fosinopril was determined to be 279 ± 18 nM (Fig. 2*A*), fosinoprilat was found to be 42 times less effective with an IC_50_ of 11.8 ± 1 μM (Fig. 2*B*), most likely due to its low membrane permeability (20). Consistent with its well-established safety profile, fosinopril had little to no activity against a panel of human cell lines (Hep G2, HeLa, HEK293, and HCT116) at concentrations up to 100 μM (Supplementary Information). The *in vitro* therapeutic index of the compound was determined to be >358, which is at least fivefold higher than current approved antiparasitic drugs (Table S1 and Fig. S5). Furthermore, a dose response relationship analysis was conducted to assess the efficacy of drug combinations involving fosinopril and either atovaquone or azithromycin against *B. duncani in vitro*. As shown in Fig. S7, fosinopril + atovaquone and fosinopril + azithromycin combinations were found to be indifferent with mean FIC_50_ values of 1, and 1.5, respectively (Fig. S7).

### Non-phosphonic acid–containing ACE-1 inhibitors lack anti-babesial activity

**Figure 3:**
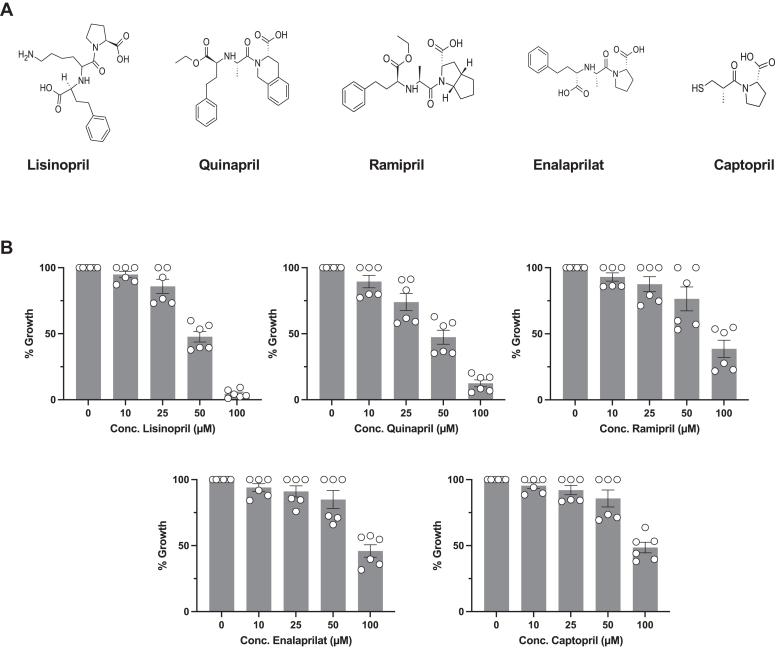
**Non-phosphonic ACE-I inhibitors: chemical structures and efficacy against *B. duncani in vitro*.***A*, chemical structures of non-phosphonic acid class ACE-I inhibitors. *B*, *B. duncani* growth as a function of the concentration of ACE-I inhibitor treatments. Each data point is the average of n = 3 biological repeats with technical duplicates ±SD.

To assess whether the antiparasitic activity of fosinopril is shared with other ACE inhibitors, the *in vitro* efficacy of the sulfhydryl-containing ACE inhibitor captopril, and the di-carboxyl-containing ACE inhibitors lisinopril, quinapril, ramipril, and enalapril were evaluated (Fig. 3*A*). *In vitro* growth inhibition assays for the five ACE inhibitors revealed moderate efficacies at best. We found a moderate inhibitory effect with lisinopril and quinapril with IC_50_ values of ∼50 μM (Fig. 3*B*). Meanwhile, the IC_50_ values of ramipril, enalaprilat, and captopril were found to be in the high micromolar range (Fig. 3*B*). No hemolytic activity was detected at concentrations of the ACE inhibitors as high as 100 μM (Fig. S8). These results suggest that the antibabesial activity of fosinopril is unique among ACE inhibitors, with phosphinate ester likely playing a critical role in its antiparasitic efficacy.

### Genetic determinant of *B. duncani* susceptibility to fosinopril

**Figure 4:**
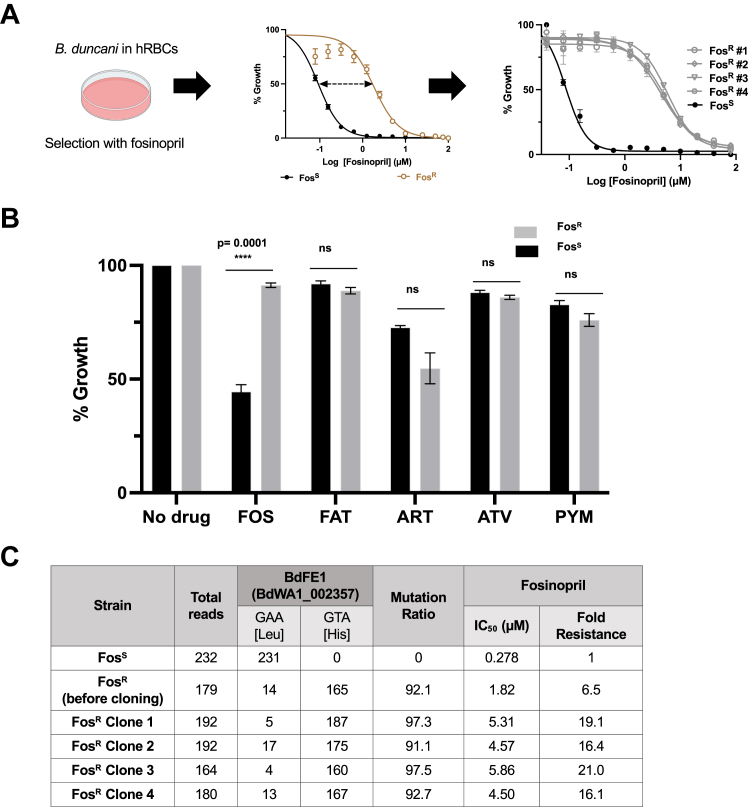
***In vitro* fosinopril-resistant phenotyping and identification of point mutation in *B. duncani BdFE1* gene.***A*, the selection of fosinopril-resistant *B. duncani* parasites using *in vitro* culture, and the response to fosinopril by fosinopril-sensitive (Fos^S^), resistant (Fos^R^) parental strain, and four resistant clones (Fos^R^ #C1-4). IC_50_ was determined as a function of strain and fosinopril concentration and compared between the resistant (Fos^R^) strains (*light brown* or *grey lines*) and the sensitive (Fos^S^) strains (*black lines*). Parasite survival was determined using a dose dependent growth inhibition assay and plotted as an average of three technical replicates (±SD). *B*, the cross-resistance profile of Fos^S^ and Fos^R^ parasites when exposed to antiparasitic drugs (FOS = fosinopril, FAT = fosinoprilat, ART = artemisinin, ATV = atovaquone, PYM = pyrimethamine) at their respective IC_50_ values. Each bar depicts the growth (measured *via* SYBR *green*) as a percentage of the treatment-free growth observed for both Fos^S^ (*black*) and Fos^R^ (*gray*) parasites. An unpaired standard *t* test was performed to determine the significance level of each drug efficacy, and the corresponding *p*-values (∗∗∗∗*p* < 0.0001) are indicated. *C*, whole-genome sequencing identified a point mutation in BdWA1_002357 (*BdFE1*), which was subsequently confirmed by Sanger sequencing in both the Fos^R^ parasite parental population and clones. The mutation ratio represents the fractions of sequenced reads containing a single nucleotide change from GAA to GTA, encoding the nonsynonymous amino acid substitution L238H. Fold resistances are the resulting fold change in IC_50_ values determined from the dose–response curves in Panel *A*.

To gain insights into the biological activity of fosinopril in *B. duncani*-infected erythrocytes, we selected for the emergence of resistant parasites in culture following treatment with 2.7 μM of the drug (10× its IC_50_) as described in Supplementary Information (21). Fosinopril-resistant parasites (Fos^R^) were selected 2 weeks post-drug treatment (2–3 weeks), cloned by limiting dilution, and their susceptibility to fosinopril was determined. As shown in Figure 4, the IC_50_ of Fos^R^ clones was found to be >16× higher than that of the isogenic parental strain (WA1 isolate) (Fig. 4*A*). The Fos^R^ clones showed no significant change in susceptibility to artemisinin, pyrimethamine or atovaquone, suggesting that the mechanism of resistance is specific to fosinopril (Fig. 4*B*). Interestingly, both the Fos^R^ clones and the parent strain (WA1) were equally susceptible to fosinoprilat (Fig. 4*B*). To map the possible genetic mutations associated with resistance to fosinopril, whole genome sequencing (WGS) was conducted on the clones (C1, C2, C3, and C4) as well as the parent strain followed by Single Nucleotide Polymorphism (SNP) analysis. Our analyses revealed a unique, nonsynonymous mutation L238H (GAA → GTA) in the predicted catalytic site encompassing the catalytic triad GxSxG in a *B. duncani* BdWA1_002357 gene encoding a putative fosinopril esterase that we named BdFE1. This substitution was identified at >90% mutation rate in the genomes BdWA1-Fos^R^ parasite clones (Fig. 4*C*). The *BdFE1* gene encodes a protein of 442 amino acids that shares ∼24% identity and ∼39% similarity with known or putative esterases from *B. microti*, *B. divergens (1802A)*, *Babesia bovis (T2Bo)*, *T. equi (WA)*, *C. parvum (Iowa II)*, and *Plasmodium falciparum* (Table S2). A residue in this catalytic region has recently been reported to be critical for the conversion of pepstatin esters to pepstatin by the *P. falciparum* PfPARE enzyme (22). Altogether these findings suggest that *B. duncani* BdFE1 is important for the activation of fosinopril into its biologically active form, fosinoprilat.

### Parasite BdFE1 converts fosinopril into fosinoprilat

**Figure 5:**
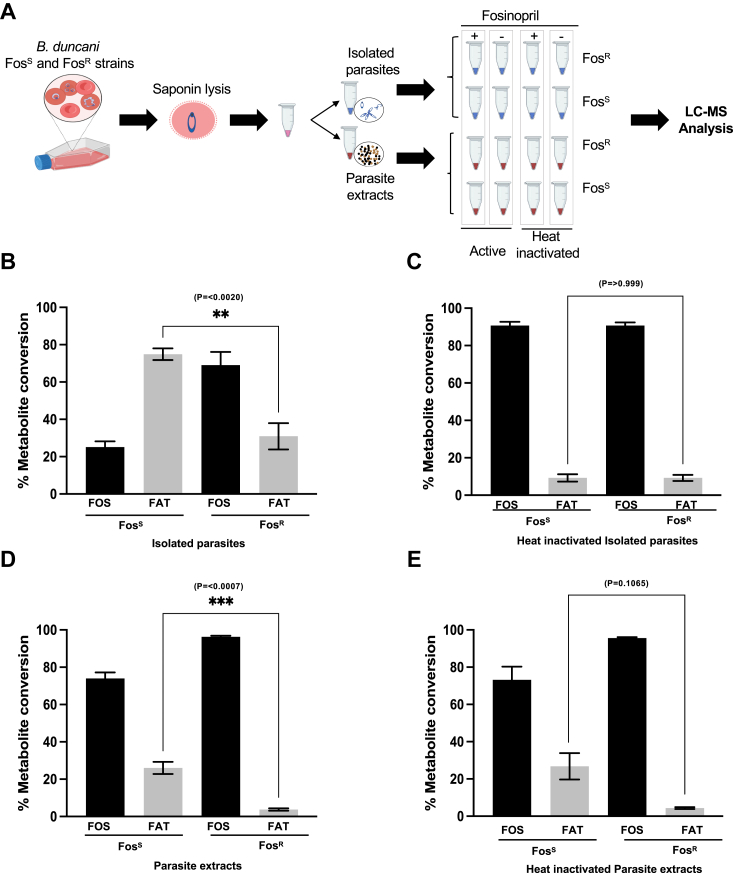
**Cellular metabolism of fosinopril in Fos**^**S**^**and Fos**^**R**^**parasites.***A*, experimental design for recovering isolated parasites and cellular extracts including heat-inactivated controls. Metabolite detection was performed using LS-MS/MS analysis. *B*, the percentage of treated fosinopril that either remained as fosinopril (FOS, *black*) or was converted to fosinoprilat (FAT, *gray*). The conversion ratio was measured after incubating either Fos^S^ (expressing wild-type BdFE1 protein) or Fos^R^ (parental or clones each expressing BdFE1^L238H^ protein) intact parasites treated with fosinopril. The difference in total fosinoprilat conversion between the Fos^S^ and Fos^R^ parasites was statistically significant (∗∗*p* < 0.02). *C*, metabolite conversion by heat-inactivated isolated intact parasites. *D*, metabolite conversion by Fos^S^ and Fos^R^ cell extracts, along with respective heat-inactivated controls. *E*, each experiment was performed twice with biological duplicates.

To further elucidate the mechanism of activation of fosinopril by *B. duncani*, isolated fosinopril-sensitive (BdWA1-Fos^S^) and -resistant (BdWA1-Fos^R^) parasites as well as parasite cellular lysates were prepared and incubated with fosinopril and the resulting metabolites were analyzed by LC-MS/MS, using appropriate standards, and quantified (Figs. 5*A* and S1). As a control, similar analyses were conducted using samples subjected to heat inactivation prior to the addition of the prodrug. The efficiency of drug processing was determined by calculating the percentage of conversion of fosinopril to fosinprilat. As shown in Figure 5*B*, ∼75% of fosinopril was converted to fosinoprilat by wild-type BdWA1-Fos^S^ isolated parasites, whereas only ∼30% conversion could be detected using BdWA1-Fos^R^ parasites harboring the BdFE1^L238H^ mutation (Fig. 5*B*). Heat-inactivated isolated parasites converted far less fosinopril to fosinoprilat as expected (approximately 7%, see Fig. 5*C*). Similarly, whereas total cell extracts from Fos^S^ parasites resulted in a 25% conversion of fosinopril to fosinoprilat, less than 5% conversion of the prodrug to the active drug could be measured using total cell extracts from Fos^R^ parasites (Fig. 5*D*). Consistent with an enzyme-mediated processing, heat inactivation of total cell extracts from both fosinopril-sensitive and Fos^R^ parasites prevented the production of fosinoprilat from fosinopril (Fig. 5*E*).

The esterase activity of the *B. duncani* fosinopril esterase BdFE1 was further examined following expression in the yeast *Saccharomyces cerevisiae* as a GST-His_6_ fusion (Fig. S2*A*). Incubation with the purified enzyme resulted in the production of free *p*-nitrophenol (*p*NP) from the substrate p-nitrophenyl-butyrate (*p*NPB) (Fig. S2*B*). No pNP formation could be detected using a heat-inactivated enzyme (Fig. S2*B*). Using the recombinant BdFE1 purified from yeast, we assessed the enzyme’s ability to convert fosinopril into fosinopriat using LC-MS/MS analysis. As shown in Fig. S2*C*, fosinopril processing by the active enzyme resulted in significant production of fosinoprilat, which was inhibited by heat-inactivation. Together, the data demonstrate that the BdFE1 esterase of *B. duncani* is essential for the conversion of fosinopril to its active form fosinoprilat, a critical modification for its antiparasitic activity.

## Discussion

This study is the first of its nature to leverage the recently developed continuous *in vitro* culture of *B. duncani* in hRBCs (8, 10, 18, 19) to screen a library of small molecules to identify new drugs to create a pipeline of new therapies for the treatment of human babesiosis. Our chemical screening, which focused on FDA-approved drugs to accelerate drug discovery *via* repurposing, has identified, fosinopril, a prodrug of the ACE inhibitor fosinoprilat, as a novel and safe compound with potent anti-babesial activity among 24 potential candidates. Thus, this study is also the first report of the anti-parasitic activity of an ACE inhibitor, a class of compounds used primarily as antihypertensive drugs. Fosinopril is a widely used drug known for its efficacy in treating hypertension and heart failure, with a well-documented history of tolerability and safety in diverse patient populations (23, 24, 25). Interestingly, no other ACE inhibitors (in either drug or prodrug forms) displayed the same anti-parasitic potency as fosinopril *in vitro*, suggesting that its activity derives from its specific structure. Consistent with its predicted low cell permeability, exogenous fosinoprilat had limited activity with an IC_50_ 42-fold higher than that of the prodrug.

**Figure 6:**
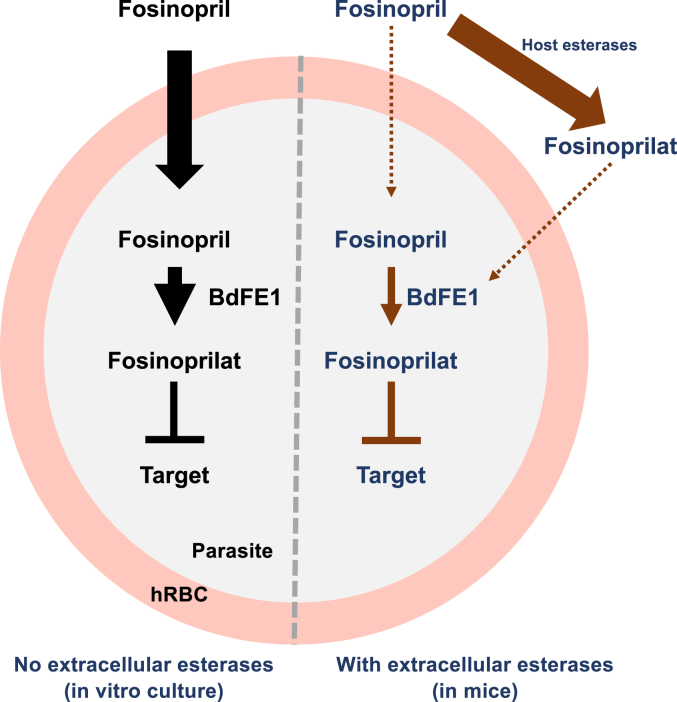
**Model for processing of fosinopril by parasite esterase and its subsequent antibabesial action.** On the *left*, in cell culture, fosinopril is transported into human red blood cells (hRBCs) and then into the parasite, whereupon processing by BdFE1 converts it into its active form, fosinoprilat. Fosinoprilat, in turn, inhibits parasite growth through as yet unknown mechanism. *In vivo* (*right panel*), host esterases convert fosinopril into fosinoprilat in the blood prior to its transport into the infected RBC. This process significantly reduces bioavailability of fosinopril to the parasite and limits its efficacy.

The finding that fosinopril possesses antiparasitic activity led us to investigate whether this activity was mediated by the molecule itself or an active derivative. Using a drug selection strategy to select Fos^R^ parasites, we identified clones that are 16-fold less susceptible to the drug than the isogenic parent wild type. The resistant Fos^R^ clones showed no cross-resistance to other unrelated drugs including artemisinin, atovaquone and pyrimethamine and had limited susceptibility to fosinoprilat, similar to the isogenic parent strain. Whole genome sequencing identified a single mutation in a new *Babesia* gene *BdFE1* encoding an enzyme belonging to the family of alpha/beta hydrolases. The mutation causes substitution of Leucine 238 with Histidine, which leads to significant changes in the protein's structure and function, potentially affecting its enzymatic activity, binding affinity, and/or stability. No paralogs of BdFE1 were found in the recently published proteome of *B. duncani*, suggesting that the enzyme could represent a possible target for the development of new antiparasitic drugs (26). BdFE1 shares homology with other esterases from *B. microti*, *B. divergens*, *B. bovis*, *T. equi*, *C. parvum*, and with the *P. falciparum* esterase enzyme recently reported to be essential for the metabolism of pepstatin esters and sesquiterpenoid esters antimalarials (22, 27, 28). Interestingly, the BdFE1 L238H mutation is in a conserved region of the predicted esterase catalytic site encompassing the mutation reported for PfPARE, which alters the susceptibility of *P. falciparum* to these drugs (Fig. S3) (22). Evidence for the role of BdFE1 in the conversion of fosinopril into its active antiparasitic moiety fosinoprilat was established by mass spectrometry analyses using both cell-based assays and purified recombinant BdFE1 enzyme. Both free isolated parasites and total cell extracts from wild type *B. duncani* actively converted fosinopril to fosinoprilat whereas only limited conversion occurred using similar sources of enzyme from Fos^R^ parasites carrying the L238H mutation. Expression of recombinant BdFE1 in heterologous systems was achieved for the wild-type version of the enzyme in the yeast *S. cerevisiae* as a GST-His_6_ tagged enzyme following cloning of the gene under the regulatory control of the strong *GAL1* promoter. The esterase activity of the purified enzyme was demonstrated using the *pNPB* substrate as measured by the production of *pNP*, and its ability to convert fosinopril to fosinoprilat was subsequently demonstrated using mass spectrometry analyses. Altogether, these genetic and biochemical assays confirm that the antibabesial activity of fosinopril is achieved following the transport of the drug into the parasite and subsequent conversion of the prodrug into its active drug fosinoprilat (Fig. 6).

While the discovery of fosinopril as an antiparasitic drug opens new avenues into the exploitation of this class of compounds and their targets for the development of new antiparasitic drugs, its limited bioavailability represents a challenge for its immediate repurposing as an antibabesial drug. In humans and mice, the compound is rapidly converted into fosinoprilat by host esterases with the half-life of the prodrug determined to be 2 to 3 h in humans and 0.5 to 1 h in mice (Fig. S4 and Table S3). Unsurprisingly, treatment of mice infected with either *B. duncani* or *B. microti* with fosinpril had little to no effect on parasitemia, whereas *ex-vivo*-treated parasites displayed reduced parasitemia and overall disease burden following injection into animals (Fig. S6). Efforts to develop analogs of fosinopril that are resistant to hydrolysis by host esterases but not parasite esterases are thus warranted.

To achieve its function in the treatment of hypertension, fosinoprilat inhibits the conversion of the decapeptide angiotensin I (proangiotensin) to the octapeptide angiotensin II, a key component of the renin-angiotensin-aldosterone system (RAAS). This dipeptidyl carboxypeptidase activity is catalyzed by ACE-1. Therefore, fosinoprilat’s antiparasitic activity could also be due to inhibition of an ACE-1-like activity that is essential for parasite survival. Although no ACE-1 homologs could be found in *Babesia* or other parasites, genome annotations identified 72 predicted proteases in *B. duncani*, 69 in *B. microti* and 140 in *P. falciparum* (PlasmoDB v. 26.0) (26, 29, 30). Identification of the main targets could help guide future structure-based drug design efforts to synthesize new fosinopril analogs or identify other classes of inhibitors that target these enzymes for the treatment of human babesiosis and possibly other parasitic infections such as malaria, toxoplasmosis, and cryptosporidiosis.

To date, there is no suitable vaccine candidate for babesiosis, and new anti-babesial drugs are needed to counter the emergence of drug-resistant parasites. The CDC-recommended drug combinations have been used with some success, but the parasites are highly tolerant to these drugs with IC_50_ values ranging between 500 nM for atovaquone and 20 μM clindamycin (1, 5, 8). Furthermore, these drugs are known to have significant side effects, (1, 31). New drugs currently in the pipeline include combinations of Endochin-Like Quinolones with atovaquone (32, 33) or tafenoquine with atovaquone (34). These new combinations require further evaluation in clinical trials. Noteworthy is the development of a continuous *in vitro* culture systems for *B. duncani* in hRBCs and reliable mouse models for *B. duncani* and *B. microti* infections (8, 10, 19). These platforms will enable effective large-scale screening of other chemical libraries as an alternative strategy to expand the anti-babesial pre-clinical development pipeline. The discovery of fosinopril as an antiparasitic drug validates this approach and portends broad implications for the accelerated discovery of novel treatments for parasitic diseases that pose global public health threats, including human malaria.

## Experimental procedures

### Materials

FDA-approved chemical library was obtained from the Yale Center for Molecular Discovery (YCMD) collection (Enzo Life Sciences). The library was comprised of 640 FDA-approved drugs seeded in 96 well plates at a 100 nl volume at a concentration of 1 mM. Fosinopril and fosinoprilat (>99% purity) and ammonium acetate (>98% purity) were purchased from Sigma-Aldrich. Water and methanol (LCMS grade) were purchased from Fisher Chemical.

### Animal studies

C3H/HeJ mice were purchased from The Jackson Laboratory. All animal experiments followed Yale University institutional guidelines for the care and use of laboratory animals under a protocol approved by the Institutional Animal Care and Use Committees.

### Primary screening of FDA-approved drug library

For drug screening, 100 μl of *B. duncani* culture (5% hematocrit and 1% starting parasitemia) were added to each well of the 96-well plate in Claycomb medium supplemented with 20% serum and containing individual compounds at 1 μM each (final DMSO concentration of 0.1%). The assay was repeated three times. After 62 h, blood smears were obtained to determine parasitemia levels as described above and SYBR Green I assay was used as described above. Percent growth for each well was obtained by comparison to the positive control drug (1 μM WR-99210) and infected control (0.1% DMSO). The data were plotted in GraphPad Prism v 9.4.1 to generate heat maps and bar graphs.

### *In vitro* drug efficacy assays

The efficacy of lead compounds on parasite growth was evaluated by studying the intra-erythrocytic development cycle (IDC) inhibition of *B. duncani* and determining the IC_50_ by the established protocol (32). Briefly, *in vitro* parasite culture (0.1% parasitemia with 5% hematocrit in complete medium) was treated with a 2-fold serially diluted concentrations of inhibitors in a 96-well plate for three 60 h. After the treatment, parasitemia was enumerated by the above-mentioned SYBR Green-I method. Background fluorescence reading obtained from uninfected RBCs in a complete medium was subtracted from each parasite-containing well. The 50% inhibitory concentration (IC_50_) of the drug was determined from the sigmoidal dose-response curve by plotting the drug log concentration against percent parasite growth using GraphPad Prism v 9.4.1. Each IC_50_ value obtained from two independent experiments with biological triplicates is shown as mean ± SD.

### WGS analysis and single-nucleotide polymorphism calling

Parasite genomic DNA was isolated using the QiAmp DNA Blood Mini kit (Qiagen cat:6950). The isolated genomic DNA was subjected to WGS on the Illumina sequencing platform at the Yale Center for Genomics Analysis (Supplementary Information).

### Analysis of fosinopril metabolism in *B. duncani*-infected RBCs by LC-MS/MS analysis

*B. duncani* parasite cultures with 15% parasitemia were collected, lysed with 0.15% saponin (Sigma: S7900) washed with PBS, and harvested parasites by centrifugation. Parasite pellets were resuspended in 20% glycerol and stored at −80 °C for further biochemical assays. These cells serve as intact parasites. The parasite crude lysate was prepared by treating the pellet with 10-fold hypotonic lysis buffer (1 mM ATP, 1 μM E64, 20 mM HEPES pH 8.0, and 0.03% SDS) and two quick freeze-thaw cycles in the liquid N_2_ followed by centrifugation of 15,000 rpm for 5 min at 4 °C. The collected supernatant was used for activity studies. For the MS analysis, equal volumes of both intact parasites and lysates and their respective heat-inactivated samples were treated with 2 μM of fosinopril, and fosinoprilat at 37 °C for 0 h and 1 h. The treated samples were further resuspended in equal volumes of cold 20 mM NaPO4, (pH 7.2) buffer for 3 h at 4 °C with intermittent mixing. The reaction mixture was further extracted with three volumes of cold acetonitrile by centrifugation at 3400 rpm at 4 °C for 15 min and the supernatant was subjected to LC-MS/MS analysis to detect the metabolite. Uninfected intact and heat-inactivated hRBCs serve as controls. The amount of fosinopril and fosinoprilat detected from both the BdWA1Fos^S^ and BdWA1Fos^R^ intact parasites and lysates were normalized with the respective treated RBC controls. The total % metabolite conversion from both was plotted by using the Graph Pad Prism v 9.4.1.

### Institutional biosafety statement

All studies involving the use of human blood and *Babesia* parasites in culture were approved by the Institutional BioSafety Committee at Yale University.

## Data availability

WGS (whole genome sequencing) illumina reads will be accessible as SRA records. SRA data: PRJNA1008687, Submission ID: SUB13791426 with following accession numbers: SRR25745272, SRR25745273, SRR25745269, SRR25745271, SRR25745268, SRR25745270. GitHuB Repository information: https://github.com/ucrbioinfo/fosinopril.

## Supporting information

This article contains supporting information.

## Conflict of interest

The authors declare that they have no conflicts of interest with the contents of this article.

## References

[1] Renard I., Ben Mamoun C. (2021). Treatment of human babesiosis: then and now. Pathogens.

[2] Karshima S.N., Karshima M.N., Ahmed M.I. (2022). Global meta-analysis on Babesia infections in human population: prevalence, distribution and species diversity. Pathog. Glob. Health.

[3] Werden L., Lindsay L.R., Barker I.K., Bowman J., Gonzales E.K., Jardine C.M. (2015). Prevalence of anaplasma phagocytophilum and Babesia microti in Ixodes scapularis from a newly established Lyme disease endemic area, the Thousand Islands region of Ontario, Canada. Vector Borne Zoonotic Dis..

[4] Madison-Antenucci S., Kramer L.D., Gebhardt L.L., Kauffman E. (2020). Emerging tick-borne diseases. Clin. Microbiol. Rev..

[5] Swanson M., Pickrel A., Williamson J., Montgomery S. (2023). Trends in reported babesiosis cases United States, 2011–2019. MMWR Morb Mortal Wkly Rep.

[6] Wormser G.P., Prasad A., Neuhaus E., Joshi S., Nowakowski J., Nelson J. (2010). Emergence of resistance to azithromycin-atovaquone in immunocompromised patients with Babesia microti infection. Clin. Infect. Dis..

[7] Simon M.S., Westblade L.F., Dziedziech A., Visone J.E., Furman R.R., Jenkins S.G. (2017). Clinical and molecular evidence of atovaquone and azithromycin resistance in relapsed Babesia microti infection associated with rituximab and chronic lymphocytic leukemia. Clin. Infect. Dis..

[8] Abraham A., Brasov I., Thekkiniath J., Kilian N., Lawres L., Gao R. (2018). Establishment of a continuous in vitro culture of Babesia duncani in human erythrocytes reveals unusually high tolerance to recommended therapies. J. Biol. Chem..

[9] Dao A.H., Eberhard M.L. (1996). Pathology of acute fatal babesiosis in hamsters experimentally infected with the WA-1 strain of Babesia. Lab. Invest..

[10] Pal A.C., Renard I., Singh P., Vydyam P., Chiu J.E., Pou S. (2022). Babesia duncani as a model organism to study the development, virulence and drug susceptibility of intraerythrocytic parasites in vitro and in vivo. J. Infect. Dis..

[11] Warner N.J., Rush J.E. (1988). Safety profiles of the angiotensin-converting enzyme inhibitors. Drugs.

[12] NCD Risk Factor Collaboration (NCD-RisC) (2021). Worldwide trends in hypertension prevalence and progress in treatment and control from 1990 to 2019: a pooled analysis of 1201 population-representative studies with 104 million participants. Lancet.

[13] Williams B., Zhang Y. (2020). Hypertension, renin-angiotensin-aldosterone system inhibition, and COVID-19. Lancet.

[14] Bernstein K.E., Ong F.S., Blackwell W.L., Shah K.H., Giani J.F., Gonzalez-Villalobos R.A. (2013). A modern understanding of the traditional and nontraditional biological functions of angiotensin-converting enzyme. Pharmacol. Rev..

[15] Skeggs L.T., Kahn J.R., Shumway N.P. (1956). The preparation and function of the hypertensin-converting enzyme. J. Exp. Med..

[16] Zheng W., Tian E., Liu Z., Zhou C., Yang P., Tian K. (2022). Small molecule angiotensin converting enzyme inhibitors: a medicinal chemistry perspective. Front. Pharmacol..

[17] Duchin K.L., Waclawski A.P., Tu J.I., Manning J., Frantz M., Willard D.A. (1991). Pharmacokinetics, safety, and pharmacologic effects of fosinopril sodium, an angiotensin-converting enzyme inhibitor in healthy subjects. J. Clin. Pharmacol..

[18] Singh P., Pal A.C., Mamoun C.B. (2022). An alternative culture medium for continuous in vitro propagation of the human pathogen Babesia duncani in human erythrocytes pathogens.

[19] Kumari V., Pal A.C., Singh P., Mamoun C.B. (2022). Babesia duncani in culture and in mouse (ICIM) model for the advancement of Babesia biology, pathogenesis, and therapy. Bio Protoc..

[20] Ranadive S.A., Chen A.X., Serajuddin A.T.M. (1992). Relative lipophilicities and structural-pharmacological considerations of various angiotensin-converting enzyme (ACE) inhibitors. Pharm. Res..

[21] Ng C.L., Fidock D.A. (2019). Plasmodium falciparum in vitro drug resistance selections and gene editing methods. Mol. Biol..

[22] Istvan E.S., Mallari J.P., Corey V.C., Dharia N.V., Marshall G.R., Winzeler E.A. (2017). Esterase mutation is a mechanism of resistance to antimalarial compounds. Nat. Commun..

[23] Murdoch D., McTavish D. (1992). Fosinopril. A review of its pharmacodynamic and pharmacokinetic properties, and therapeutic potential in essential hypertension. Drugs.

[24] Shionoiri H., Naruse M., Minamisawa K., Ueda S., Himeno H., Hiroto S. (1997). Fosinopril. Clinical pharmacokinetics and clinical potential. Clin. Pharmacokinet..

[25] Davis R., Coukell A., McTavish D. (1997). Fosinopril. A review of its pharmacology and clinical efficacy in the management of heart failure. Drugs.

[26] Singh P., Lonardi S., Liang Q., Vydyam P., Khabirova E., Fang T. (2023). Babesia duncani multi-omics identifies virulence factors and drug targets. Nat. Microbiol..

[27] Butler J.H., Baptista R.P., Valenciano A.L., Zhou B., Kissinger J.C., Tumwebaze P.K. (2020). Resistance to some but not other dimeric lindenane sesquiterpenoid esters is mediated by mutations in a Plasmodium falciparum esterase. ACS Infect. Dis..

[28] Sindhe K.M.V., Wu W., Legac J., Zhang Y.-K., Easom E.E., Cooper R.A. (2020). Plasmodium falciparum resistance to a lead benzoxaborole due to blocked compound activation and altered ubiquitination or sumoylation. mBio.

[29] Cornillot E., Hadj-Kaddour K., Dassouli A., Noel B., Ranwez V., Vacherie B. (2012). Sequencing of the smallest Apicomplexan genome from the human pathogen Babesia microti. Nucleic Acids Res..

[30] Wang J., Chen K., Yang J., Zhang S., Li Y., Liu G. (2022). Comparative genomic analysis of Babesia duncani responsible for human babesiosis. BMC Biol..

[31] Krause P.J., Gewurz B.E., Hill D., Marty F.M., Vannier E., Foppa I.M. (2008). Persistent and relapsing babesiosis in immunocompromised patients. Clin. Infect. Dis..

[32] Chiu J.E., Renard I., Pal A.C., Singh P., Vydyam P., Thekkiniath J. (2021). Effective therapy targeting cytochrome bc(1) prevents Babesia erythrocytic development and protects from lethal infection. Antimicrob. Agents Chemother..

[33] Lawres L.A., Garg A., Kumar V., Bruzual I., Forquer I.P., Renard I. (2016). Radical cure of experimental babesiosis in immunodeficient mice using a combination of an endochin-like quinolone and atovaquone. J. Exp. Med..

[34] Vydyam P., Pal A.C., Renards I., Chand M., Kumari V., Gennaro J.C. (2023). Tafenoquine-atovaquone combination achieves radical cure and confers sterile immunity in experimental models of human Babesiosis. J. Infect. Dis..

