# Supplementary material for: Babesia BdFE1 esterase is required for the anti-parasitic activity of the ACE inhibitor fosinopril
Source: J Biol Chem. 2023 Oct 4;299(11):105313. doi: 10.1016/j.jbc.2023.105313 (PMC10663679; doi:10.1016/j.jbc.2023.105313)
Supplement: Supplemental Figures S1–S8 [file mmc1.docx]

**Supporting Information Figures and Legends:**

**Fig. S1. Detection of cellular fosinopril and fosinoprilat by LC-MS/MS. A)** *B. duncani* isolated parasites were incubated with fosinopril, harvested, and extracts were prepared for LC-MS/MS analysis. The presence or absence of both fosinopril and fosinoprilat were determined by recording their appropriate mass and retention times. The corresponding dominant peaks of both compounds in the spectra were identified as shown. Subsequent quantitative analyses of calibration curves were conducted using LC-MS/MS. **B)** Spectra obtained from the LC-MS/MS analysis of the heat-inactivated isolated parasites are displayed for comparison. **C)** Spectra acquired from the LC-MS/MS analysis of intact parasite extracts. **D)** Spectra obtained from the LC-MS/MS analysis of heat-inactivated parasite extracts. Images shown are representative; each experiment was performed twice with technical duplicates.

**Fig. S2. Expression of recombinant BdFE1 and its esterase activity. A)** Recombinant His_6_-BdFE1-GST protein was expressed in yeast and detected in yeast total extracts using an anti-histidine monoclonal antibody. The two left lanes correspond to proteins from cells harboring the empty vector (W303-1B/pESC-URA), both in glucose- (repression of GAL1 promoter) and in galactose- (induction of GAL1 promoter) based media. The right two lanes correspond to cells expressing BdFE1 (W303-1B/pESC-BdFE1-URA) on glucose or galactose. The purified BdFE1 protein with both GST and His tags with predicted molecular weight of ~77 kDa was detected using the HRP-conjugated α-His_6_ antibody. **B)** The esterase activity of purified recombinant BdFE1 protein (brown) on *p*-nitrophenyl butyrate (*p*NPB) was compared to that of proteins isolated from cells harboring the empty vector (black) or buffer (circle). Increasing concentrations of *p*NPB were incubated with the purified BdFE1, and the formation of the product *p*-nitrophenol (PNP) was monitored at 400 nm. The amount of *p*PNP generated was plotted as a function of *p*NPB concentration and fitted to a Michaelis-Menten equation. The activity of the wild-type protein (brown line) was found to be significantly higher than that of the empty vector proteins (black) and the buffer (black dashed lines) based on a student t-test. The error bars on the graph represent the standard deviation (±SD) from the mean of three biological replicates. **C)** BdFE1-mediated conversion of fosinopril to fosinoprilat. Intact or heat-inactivated BdFE1 proteins were incubated with fosinopril, and the conversion rate from fosinopril to fosinoprilat was determined using LC-MS/MS. Statistical analyses were conducted for each experiment to determine significance. Values are the average of two experiments ±SD.

**Fig. S3. Multiple sequence alignment of the BdFE1 protein.** Sequence alignment of BdFE1 and several orthologs from different protozoan parasites using the Clustal Omega server. Similar amino acid residues are highlighted in blue, while completely identical residues are colored in red. The catalytic triad GxSxG, which is essential for catalysis, is highlighted. Leucine 238 in BdFE1 is marked by the arrow.

**Fig. S4.** **Pharmacokinetics of fosinopril in mice.** Concentration of fosinopril in mouse serum 2 hours post intravenous administration (I.V, black lines, t_1/2_=52 min) at a dose of 10 mg/kg or intraperitoneal administration (I.P, purple lines, t_1/2_=30 min) at a dose of 30 mg/kg body weight. Data points represent averages of serum levels in mice ± SD.

**Fig. S5. In vitro efficacy of quinine, azithromycine, atovaquone and clindamycin against *Babesia duncani***. Growth of *B. duncani*-infected RBCs in DMEM-F12-based complete medium the absence or presence of increasing concentrations of the drugs was determined using the SYBR Green-I assay. The data were used to generate the curves in A-D and to calculate the IC_50_ values for each drug using GraphPad Prism software. **A)** AZT = Azithromycin, **B)** CLN = Clindamycin, **C)** ATV = Atovaquone, and **D)** QUI = Quinine. Data are derived from two independent biological experiments with three technical replicates each and are expressed as the mean ± SD.

**Fig. S6. In vivo and ex-vivo efficacy of fosinopril in a mouse model of lethal *B. duncani* infection**. **A)** Female C3H/HeJ mice were injected intravenously with 10^3^ *B. duncani*-infected red blood cells. Animals were treated daily (DPI 1 to 10) by oral gavage with the vehicle alone (PEG400), fosinopril at 10 mg/kg (blue line), 30 mg/kg (purple line) or 100 mg/kg (red line). **B)** Female C3H/HeJ mice were injected intravenously with 10^3^ *B. duncani*-infected red blood cells cultured *in vitro* in human erythrocytes in the absence or presence of fosinopril at 10X IC_50_ (2.7 µM) for 24 hours. **C-D)** Survival rates of *B. duncani*-infected mice from A and B. E: indicates when mice were euthanized.

**Fig. S7.** **Dose-response analysis of combinations of fosinopril with either atovaquone or azithromycin against *B. duncani*.** **A-B)** Dose-response relationship for each drug and for the drug combinations were conducted using 9 fixed ratios of fosinopril and either atovaquone or azithromycin, and IC_50_ values were calculated for each combination. The FIC data for the 50% inhibitory concentration are averaged from two independent experiments, each with biological triplicates. FIC values that closely align with the additive line (a dotted line drawn between the individual drug effects). The calculated FIC values from each combination were used to generate the isobolograms. FIC < 0.5 indicates synergism, > 0.5–1 additive effects, > 1 to < 2 indifference, and ≥ 2 antagonism.

**Fig. S8. ACE inhibitors lack hemolytic activity.** Graphs depict total hemoglobin released from human RBCs in the absence or presence of increasing concentrations of various ACE inhibitors (**A**=100 µM, **B**=50 µM, **C**=25 µM, and **D**=10 µM). Released hemoglobin in the culture medium was measured using a BioTek Synergy™ Mx spectrophotometer at 527 nm. RBCs treated with culture media, both with and without DMSO, or treated with 1% saponin were used as controls.
