# Supplementary material for: Babesia BdFE1 esterase is required for the anti-parasitic activity of the ACE inhibitor fosinopril
Source: J Biol Chem. 2023 Oct 4;299(11):105313. doi: 10.1016/j.jbc.2023.105313 (PMC10663679; doi:10.1016/j.jbc.2023.105313)
Supplement: Supplemental Table S1 [file mmc2.docx]

**Table S1: Cytotoxicity of fosinopril and atovaquone on human cell lines**

**
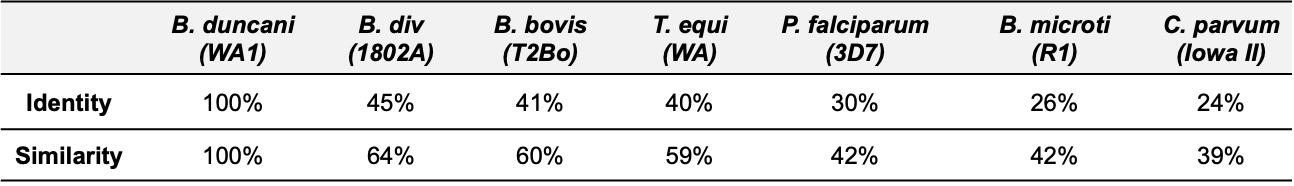

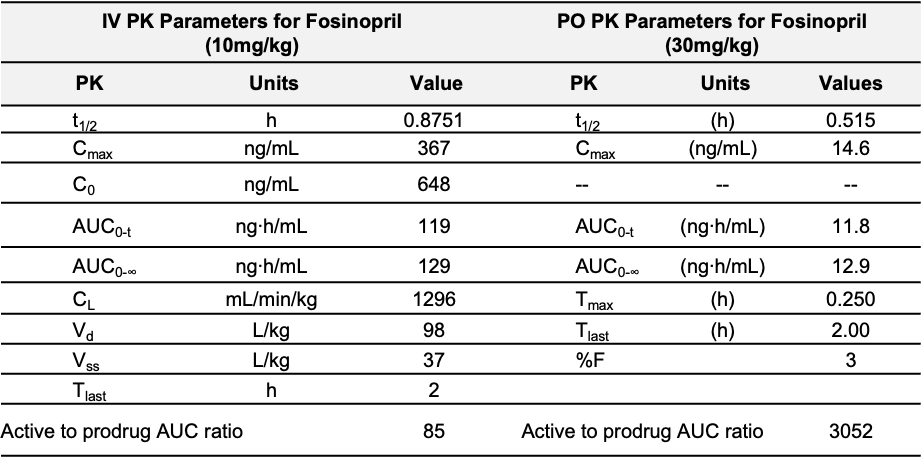
**

**Table S2: Protein sequence identity and similarity %**

**Table S3: Pharmacokinetic properties of Fosinopril in mice**
