# Supplementary material for: Babesia BdFE1 esterase is required for the anti-parasitic activity of the ACE inhibitor fosinopril
Source: J Biol Chem. 2023 Oct 4;299(11):105313. doi: 10.1016/j.jbc.2023.105313 (PMC10663679; doi:10.1016/j.jbc.2023.105313)
Supplement: Supporting Information Text [file mmc3.docx]

**Supporting Information Text for:**

*Babesia* BdFE1 Esterase is Required for the Anti-parasitic Activity of the ACE Inhibitor Fosinopril

Pratap Vydyam^1^, Jae-Yeon Choi^1^, Shalev Gihaz^1^, Meenal Chand^1^, Meital Gewirtz^1^, Jose Thekkiniath^1^, Stefano Lonardi^2^, Joseph C. Gennaro^1^, and Choukri Ben Mamoun^1*^

^1^Department of Internal Medicine, Section of Infectious Diseases, Yale School of Medicine, New Haven, CT 06520, USA.

^2^Department of Computer Science and Engineering, University of California, Riverside, CA, USA

*Correspondence and requests for materials should be addressed to: [choukri.benmamoun@yale.edu](mailto:choukri.benmamoun@yale.edu)

**Supporting text:**

**Parasite strains and culture conditions.** The *B. duncani* parasite strain WA1 was used for this research. It was obtained from BEI resources and is maintained continuously in our lab in human red blood cells *in vitro* as previously described (1). For regular maintenance of the parasite, *B. duncani*-infected red blood cells were seeded at 1% parasitemia and 5% hematocrit using human RBCs (A^+^) in DMEM-F12-based complete medium (supplemented with 20% fetal bovine serum, hypoxanthine thymidine supplement (100 µM) (Sigma H0137) 50x, Glutamine Plus (Atlanta Biologicals B90210), Antibiotic Antimycotic (Corning 30004CI), 10mg/mL Gentamycin Reagent Solution (Gibco 15710-064)). Cultures were incubated in a modulator-incubator chamber with a gas mixture of 2% O_2_, 5% CO_2_, and 93% N_2_ at 37°C.

**Evaluation of drug cytotoxicity.** The HeLa, HepG2, HEK, and HCT116 cell lines were procured from the American Type Culture Collection (ATCC) and were cultured in Dulbecco’s Modified Eagle’s Medium (DMEM) (Invitrogen 11995-065) supplemented with 25 mM glucose, 1 mM sodium pyruvate, 5 mM HEPES, 10% fetal bovine serum (FBS), and penicillin-streptomycin (50 U/mL penicillin, 50 µg/mL streptomycin). For cytotoxicity assessments, 20,000 cells were seeded in each well of 96-well tissue culture plates and allowed to adhere for 24 hours. Subsequently, the cells were treated with varying concentrations of drugs using a 2-fold serial dilution approach. Negative and positive vehicle controls were established by adding 0.1% and 10% DMSO to specific wells, respectively. Incubation of the plates was carried out at 37˚C for 48 hours. Following drug treatment, each well was exposed to 0.5 mg/mL of MTT reagent (M6494) for 4 hours in the absence of light at 37˚C. The formazan crystals produced by viable cells were solubilized by adding 100 µL of dimethyl sulfoxide (DMSO) to each well. The absorbance was then measured at 590 nm using the BioTek Synergy™ Mx Microplate Reader. The percentage of cell viability was calculated by normalizing the absorbance values to the mean of the wells with 10% DMSO (set as 100% toxicity) and the mean of the vehicle control wells (set as 0% toxicity). The half-maximal inhibitory concentration (IC_50_) was determined through nonlinear regression dose-response curve analysis, with data plotted using GraphPad Prism v 9.4.1.

**Determination of parasitemia**. Thin blood smears were stained using Giemsa stain. A minimum of 3000 RBCs per slide were examined at 1,000x magnification to determine the percentage of parasitemia. To assess growth, the SYBR Green I assay was employed. In this assay, 100 µL of culture from each well of the 96-well plate was transferred to a Costar 96-well black bottom plate. These aliquots were thoroughly mixed with an equivalent volume of SYBR Green lysis buffer composed of 20 mM Tris (pH 7.4), 5 mM EDTA, 0.008% saponin, 0.08% Triton X-100, and 1x SYBR™ Green I (S7567). Subsequently, the Costar plates were incubated in darkness at room temperature for 1 hour. Fluorescence measurements were obtained using a BioTek Synergy™ Mx fluorescence plate reader, with excitation set at 480 nm and emission recorded at 540 nm.

**Selection of drug resistant parasites *in vitro*.** The process of selecting drug-resistant parasites was carried out by adapting the single-step selection protocol employed in *P. falciparum* (2-4). In brief, a culture consisting of 2 × 10^9^ red blood cells (RBCs) infected with *B. duncani*, with a hematocrit level of 4%, was maintained in the presence of an inhibitor at a concentration 10 times higher than the IC_50_ and incubated at 37°C. To ensure the continual presence of the inhibitor, we refreshed the culture daily with new media containing the drug, all the while monitoring parasitemia over time using Giemsa staining. The selection process continued until no parasites could be detected. Following this initial phase, we transferred the cultures to a drug-free medium, changing the medium every 2 days, and adding fresh RBCs (at 0.5% hematocrit) every 7 days. These conditions were maintained until parasites were detectable by light microscopy. Four resistant clones were selected by limiting dilution (5) from the parasite population and their drug susceptibility determined using the SYBR Green-I-based growth inhibition assay. Untreated parental parasites were harvested at the beginning of the drug selection for genomic DNA isolation to be used as a control following whole genome sequencing.

**Whole Genome Sequencing analysis and Single Nucleotide Polymorphism calling.** Genomic DNA from cloned parasites was subjected to Whole Genome Sequencing (WGS) using the Illumina sequencing platform at the Yale Center for Genomics Analysis (YCGA). In summary, 3 µg of the isolated genomic DNA was first sheared to achieve a 500 bp size. These fragments were subsequently sequenced on an Illumina MiSeq flow cell, generating 150 bp paired-end reads with a coverage depth of 50x. WGS analysis and SNP calling were performed using Genious Prime software (version 6) (available at [https://www.geneious.com](https://www.geneious.com/)). Raw reads underwent trimming via the BB Duck trimming tool, and pairing of both reads was ensured. These paired Illumina reads were aligned to the reference *B. duncani* WA1 genome with medium sensitivity, fine-tuning involving a minimum of five iterations. Reads that failed to map to the reference genome were excluded from further analysis. Mapped reads were used for SNP calling, with a minimum coverage threshold of 100 and a minimum variant frequency of 0.25 applied. This facilitated the identification of high-confidence SNPs present in drug-resistant parasites. SNPs exhibiting a variant frequency exceeding 80% were compared to the *B. duncani* WA1 parental genome were considered as potential candidates for drug resistance-related mutations. A set of SNPs unique to different clones but not shared with the parent strain (WA1 isolate) were computed using custom Python scripts.

**Pharmacokinetic study of fosinopril.** Pharmacokinetic studies involving fosinopril in mice were conducted in collaboration with Jubilant ([https://www.jubilantindustries.com](https://www.jubilantindustries.com/)). In brief, fosinopril was administered to 24 male BALB/c mice at doses of 30 mg/kg orally and 10 mg/kg intravenously (PO: dissolved in a mixture of tween 80 [0.5%] and methylcellulose [99.5% v/v]; IV: normal saline). Blood samples were collected from both the control and treated mice at specified time intervals over a 24-hour period. The samples were collected in K_2_EDTA tubes and subsequently separated into plasma for downstream analysis. The sample processing involved the addition of 50 µL of plasma to 400 µL of 100% methanol containing an internal standard (Diclofenac-100 ng/mL). After thorough vortexing for 1 minute, the samples were centrifuged at 14,000 rpm for 5 minutes at 4°C. An aliquot of the resulting supernatant was separated, and 10 µL was injected into the LC-MS/MS system for the quantification of fosinopril and fosinoprilat concentrations. To prepare the working stock solutions of fosinopril and fosinoprilat, 5 µL of each compound was spiked into 45 µL of blank plasma to achieve the desired concentrations.

**Expression and purification of recombinant BdFE1from yeast.** Codon-optimized BdFE1 and BdFE1^L238H^ genes were synthesized and cloned into pESC-URA vector (GenScript) under the control of *GAL1* promoter. The resulting proteins contain N-terminal GST and C-terminal His_6_ tags. The plasmids were transformed into a yeast (W303-1B) strain using yeastmaker™ yeast transformation system 2 (Takara Bio USA Inc, Cat# 630439). Transformants were selected by picking the colonies grown on the plate containing synthetic uracil dropout medium supplemented with 2% glucose. The cells harboring the plasmid constructs (W303-1B/pESC-URA-BdFE1 and W303-1B/pESC-URA-BdFE1^L238H^) were pre-grown in the SC minimal liquid medium supplemented with 2% glycerol as a carbon source at 30 ˚C and grown to the mid-log phase. The cells were harvested and re-suspended in SC minimal liquid medium supplemented with 2% galactose at the OD_600_ of 0.5 and grown for 4 hr at 30 ˚C in the shaking incubator to induce protein expression. Protein purification was carried out as reported earlier (6) with minor modifications. Briefly, the cells were harvested by centrifugation at 2500 g for 5 min at 4 ˚C, washed once with water, and cell pellets were resuspended in the lysis buffer (50 mM Phosphate buffer pH 8, 200 mM NaCl, 1% Triton X-100, 10 mM Imidazole, 1x Protease inhibitor cocktail) and the cell suspension was transferred into the Eppendorf tube containing (50 ml per 2000 OD_600_ cells) containing 0.5 mm-diameter glass beads at 300 µL equivalent volume. The cell-free extracts were obtained by bead beating for a total of 7 cycles of vortexing for 30 sec and pause on ice for 1 minute, followed by centrifugation at 3,000 g for 5 min. The recombinant proteins were purified on Ni-NTA agarose (Qiagen# 30210) after dilution in the 5 mL of the binding buffer (50 mM Phosphate buffer pH 8, 200 mM NaCl, 1% Triton X-100, 20 mM Imidazole) and incubation with the agarose for 1 hour at 4 ˚C. The column was washed with 5 ml of binding buffer 3 times. The His_6_-tagged recombinant proteins were eluted with the elution buffer (50 mM Phosphate buffer, pH 8, 200 mM NaCl, and 300 mM Imidazole). In total, 3 fractions of 0.5 ml each were collected. The fractions containing the recombinant BdFE1s were analyzed by western blot using an HRP-conjugated anti-His_6_ antibody. Esterase activity assay was performed as described previously (7). Briefly, purified BdFE1 was incubated with serially diluted *p*NPB substrate (0 to 1000 mM in PBS) for 5 min at room temperature. The reactions were stopped by adding an equal volume of acetonitrile and the hydrolyzed yellow product *p*-nitrophenol was recorded at 400 nM. Total protein extracts from cells harboring an empty vector (W303-1B/pESC-URA) were used as the control. To monitor fosinopril processing by BdEF1, the purified protein was incubated with 2 µM of fosinopril at 37˚C for 1h. Metabolites were subsequently extracted using acetonitrile, and the samples were sent for metabolite detection through LC-MS/MS, as mentioned above. Heat-inactivated protein samples and DMSO-treated samples were used as controls.

***In vitro* hemolysis analysis.** Red blood cell (RBC) lysis in the presence of ACE inhibitors was investigated as follows: ACE inhibitors at concentrations of 10, 25, 50 and 100 were added to intact human RBCs and cells were incubated at 37˚C for 64 hours. After incubation, released hemoglobin in the supernatant was quantified by measuring the absorbance at 527 nm. Treatment of human RBCs with 1% saponin-served as a positive control for total hemolysis. Human RBCs treated with either culture media or DMSO were used as negative controls. The total released hemoglobin from each sample was plotted and represented using GraphPad Prism.

**Efficacy of fosinopril-atovaquone and fosinopril-azithromycin combinations and isobologram analysis.** The effectiveness of drug combinations was assessed through a modified fixed ratio isobologram analysis method, as previously detailed (8). Briefly, stocks of eight-fold IC_50_ concentrations of fosinopril and antiparasitic drugs atovaquone and azithromycin were initially mixed in 9 ratios (1:9, 2:8, 3:7, 4:6, 5:5, 6:4, 7:3, 8:2, 9:1) and were subsequently subjected to a two-fold serial dilution in a culture medium. Parasites (at 5% hematocrit and 0.2% parasitemia) were then exposed to each of these drug combinations for 64 hours. For each specific drug combination, we determined the IC_50_ values, which were subsequently used to calculate the fractional inhibitory concentration (FIC) for each drug, employing the standard equation:

**FIC = IC_50_ of the drug in combination / IC_50_ of the drug alone.**

The mode of drug interaction was determined from the isobologram, which was plotted using the FIC values of both drugs in GraphPad Prism. FIC < 0.5 indicates synergism, > 0.5–1 additive effects, > 1 to < 2 indifference, and ≥ 2 antagonism (9).

**Supplementary Information References:**

1. Abraham, A., Brasov, I., Thekkiniath, J., Kilian, N., Lawres, L., Gao, R. *et al.* (2018) Establishment of a continuous in vitro culture of Babesia duncani in human erythrocytes reveals unusually high tolerance to recommended therapies J Biol Chem **293**, 19974-19981 10.1074/jbc.AC118.005771

2. Ng, C. L., andFidock, D. A. (2019) Plasmodium falciparum In Vitro Drug Resistance Selections and Gene Editing Methods Mol Biol **2013**, 123-140 10.1007/978-1-4939-9550-9_9

3. Ross, L. S., Gamo, F. J., Lafuente-Monasterio, M. J., Singh, O. M., Rowland, P., Wiegand, R. C. *et al.* (2014) In vitro resistance selections for Plasmodium falciparum dihydroorotate dehydrogenase inhibitors give mutants with multiple point mutations in the drug-binding site and altered growth J Biol Chem **289**, 17980-17995 10.1074/jbc.M114.558353

4. Corey, V. C., Lukens, A. K., Istvan, E. S., Lee, M. C. S., Franco, V., Magistrado, P. *et al.* (2016) A broad analysis of resistance development in the malaria parasite Nat Commun **7**, 11901 10.1038/ncomms11901

5. Butterworth, A. S., Robertson, A. J., Ho, M. F., Gatton, M. L., McCarthy, J. S., andTrenholme, K. R. (2011) An improved method for undertaking limiting dilution assays for in vitro cloning of Plasmodium falciparum parasites Malar J **10**, 95 10.1186/1475-2875-10-95

6. Gihaz, S., Gareiss, P., Choi, J. Y., Renard, I., Pal, A. C., Surovsteva, Y. *et al.* (2022) High-resolution crystal structure and chemical screening reveal pantothenate kinase as a new target for antifungal development Structure **30**, 1494-1507.e1496 10.1016/j.str.2022.09.001

7. Istvan, E. S., Mallari, J. P., Corey, V. C., Dharia, N. V., Marshall, G. R., Winzeler, E. A. *et al.* (2017) Esterase mutation is a mechanism of resistance to antimalarial compounds Nat Commun **8**, 14240 10.1038/ncomms14240

8. Chiu, J. E., Renard, I., Pal, A. C., Singh, P., Vydyam, P., Thekkiniath, J. *et al.* (2021) Effective Therapy Targeting Cytochrome bc(1) Prevents Babesia Erythrocytic Development and Protects from Lethal Infection Antimicrob Agents Chemother Aac0066221 10.1128/aac.00662-21

9. Singh, A. P., Preet, S., andRishi, P. (2011) Augmentation of antimicrobial activity of conventional antibiotics by cell-free extract of L. plantarum The Journal of Antibiotics **64**, 795-798 10.1038/ja.2011.92
